# Metropolitan Sanitation

**Published:** 1906-01-27

**Authors:** 


### The Hospital.

A Journal of

The Medical Sciences and Hospital Administration.

Vol. XXXIX.—No. 1,009. SATURDAY, JANUARY 27, 1906.

### Metropolitan Sanitation.

The issue of the annual report of the Public Health Committee of the London County Council, submitting the report of the Medical Officer of Health of the county, and important appendices thereto, is every year a timely reminder of the extent and value of the work accomplished by the Council and its officers in directions scarcely at all open to the public view, and hence liable to be left almost unnoticed alike by its critics and by its admirers. It is none the less true that to work of this description, unobtrusively performed and of no obvious political significance, the inhabitants of the metropolis are indebted for the maintenance of the conditions which render it a safe and even a salubrious place of habitation. The people of London, generally speaking, have no conception of the watchful care which is exercised over their welfare and over the removal of all conditions by which it could be prejudicially affected. Our limits forbid more than an almost casual reference to the number and variety of the acts and inquiries falling under this head, and it may be a sufficient illustration to say that in the year 1904, 34,488 workshops in London were under the supervision of the local authorities, and that 18,922 conditions requiring remedy were discovered and dealt with during the year.

The considerable time required for bringing together all the materials of the report, which necessarily embraces the proceedings of many different branches and departments, renders it apparently somewhat belated; so that now, in the beginning of 1906, it is the doings and the statistics of 1904 which are brought under public notice. It follows that evils may be described for which effective remedies have since been brought into operation, and that to this extent the report may represent only past circumstances; but, on the other hand, it may not be wholly a disadvantage to be able to see the extent to which improvements have kept pace with necessities, and the manner in which the Council, by additions to its General Powers Acts, has from time to time obtained legislative authority for the control of abuses or dangers as they have successively been brought to light. The Act of 1904, for example, gave increased powers for dealing with filthy, dangerous, or unwholesome articles, with verminous houses, with fixed ashpits, and with other nuisances of various kinds.

The actual metropolitan health report of 1904 cannot be said to compare favourably with that of the preceding year. The metropolitan death-rate for the year was 16.1 per 1,000, against 15.2 in 1903; and the infantile death-rate increased from 130 per 1,000 births in 1903 to 144 per 1,000 in 1904. The marriage-rate declined from 17.5 per 1,000 persons living in 1903 to 17.0 per 1,000 in 1904; and the birth-rate for the latter year (27.9 for 1,000 persons living) is the lowest recorded since the institution of civil registration. Together with this actual and prospective decline of population, there were 489 cases of small-pox in the metropolis, with 25 deaths; 2,256 deaths from measles, and 365 from scarlet fever. The notified cases of diphtheria amounted to 7,219, and the deaths, chiefly in children of very tender age, to 723; but it is probable that this rate of mortality has since been still farther reduced. The reports of the Metropolitan Asylums Board must be held to show conclusively that danger to life from diphtheria is practically non-existent when anti-toxin is employed within twenty-four hours of the appearance of recognisable symptoms; and it is certain that knowledge of this will every year become more general, and will lead to the early notification of all suspected cases. A substantial reduction is announced both in the total number of cases of enteric fever and in the case mortality arising from them, and various researches into causes have been set on foot. The medical officer points out that some London districts suffer from this disease practically twice as much as others, and he thinks this is more especially due to the fact that the habits of the population in poor districts afford increased opportunity for the spread of the disease from one person to another. Shellfish, fried fish, and watercress have in many cases come under suspicion as infecting agents, and the condition of watercress (or, as it might sometimes be called, sewage-cress) beds has been made the subject of special investigation by the Council's medical officer and chemist, with the result that in certain instances many causes of pollution were found. In this relation, too, it must not be forgotten that the character of direct personal infectivity can no longer be denied to enteric fever with the certainty which was once thought to be attainable.

Taken as a whole, the sanitary report of the Council indicates the existence of a manifest desire, on the part of that body, to do its best for the health of the vast community committed to its care, and certainly displays no indication of any inclination to shirk its duties in this respect, such as has too often been apparent in large municipalities outside the metropolis. From this point of view we may reasonably hope that the London County Councillors who, in considerable numbers, have found seats in the new House of Commons, will be likely to form therein the nucleus of a party to which the protection of public health will present itself as an object to be as far as possible secured, or, at all events, not to be rendered subordinate to other and far less important considerations. The politicians of the past, to use a figure of Bishop Earle's, have put their feet into sanitary legislation "tenderly, like a cat into water," and the time has surely come at which the value of many permissive enactments has been so completely displayed as to afford ample ground for rendering them compulsory. The medical inspectors of the Local Government Board will have no difficulty in indicating to their chief a sufficient number of directions in which slight changes or amplifications of the law would be of signal public advantage; and the chief himself could have no more worthy ambition than that of saying that he had lifted the burden of even one prevalent disease from the shoulders of the English people. Such a work would be even greater than that of Augustus, who found Rome brick, and left it marble.

